# Global disease burden of pathogens in animal source foods, 2010

**DOI:** 10.1371/journal.pone.0216545

**Published:** 2019-06-06

**Authors:** Min Li, Arie H. Havelaar, Sandra Hoffmann, Tine Hald, Martyn D. Kirk, Paul R. Torgerson, Brecht Devleesschauwer

**Affiliations:** 1 Feed the Future Innovation Lab for Livestock Systems, Emerging Pathogens Institute, Institute for Sustainable Food Systems, Department of Animal Sciences, University of Florida, Gainesville, FL, United States of America; 2 Economic Research Service, US Department of Agriculture, Washington DC, United States of America; 3 Unit for Genomic Epidemiology, Danish Technical University, Lyngby, Denmark; 4 Research School of Population Health, The Australian National University, Canberra, Australia; 5 Section of Epidemiology, Vetsuisse Faculty, University of Zurich, Zurich, Switzerland; 6 Department of Epidemiology and Public Health, Sciensano, Brussels, Belgium; 7 Department of Veterinary Public Health and Food Safety, Faculty of Veterinary Medicine, Ghent University, Merelbeke, Belgium; Cornell University, UNITED STATES

## Abstract

Animal source foods (ASF) such as dairy, eggs, fish and meat are an important source of high-quality nutrients. Lack of ASF in diets can result in developmental disorders including stunting, anemia, poor cognitive and motor development. ASF are more effective in preventing stunting than other foods and promoting ASF consumption in low- and middle-income countries could help improve health, particularly among pregnant women and young children. Production and consumption of ASF are, however, also associated with potential food safety risks. Strengthening of food control systems, informed by quantitative assessments of the disease burden associated with ASF is necessary to meet global nutrition goals. We present the human disease burden associated with 13 pathogens (bacteria and parasites) in ASF, based on an analysis of global burden of foodborne disease (FBD) estimates of the WHO Foodborne Disease Burden Epidemiology Reference Group (FERG). The FBD burden of these pathogens was combined with estimates of the proportion of disease transmitted by eight main groups of ASF. Uncertainty in all estimates was accounted for by Monte Carlo simulation. In 2010, the global burden of ASF was 168 (95% uncertainty interval (UI 137–219) Disability Adjusted Life Years (DALYs) per 100,000 population, which is approximately 35% of the estimated total burden of FBD. Main pathogens contributing to this burden included non-typhoidal *Salmonella enterica*, *Taenia solium*, and *Campylobacter* spp. The proportion of FBD burden associated with ASF varied considerably between subregions and between countries within subregions. Likewise, the contribution of different pathogens and ASF groups varied strongly between subregions. Pathogens with a localized distribution included *T*. *solium* and fishborne trematodes. Pathogens with a global distribution included non-typhoidal *S*. *enterica*, *Campylobacter* spp., *Toxoplasma gondii*, and *Mycobacterium bovis*. Control methods exist for many hazards associated with ASF, and their implementation is linked to economic development and effective food safety systems.

## Introduction

Animal source foods (ASF) are an important source of high-quality nutrients, including proteins, vitamins (A, D3, only source of B12), iron, zinc, calcium and folic acid. Lack of ASF in diets can result in developmental disorders including stunting, anemia, poor cognitive and motor development [1, 2]. ASF is more effective in preventing stunting than other foods or supplements [3] and promoting consumption of ASF in low- and middle-income countries is seen as important to improving the nutrition and health of vulnerable populations in these countries, in particular the health of pregnant women and young children [3–5].

Consumption of ASF are, however, also a leading point of exposure to foodborne pathogens [6–9]. Foodborne disease can impede or undo the nutritional contribution of ASF and other nutritious foods. Because of this, the Second International Conference on Nutrition 2014 recognized the importance of food safety as an enabling condition for improving malnutrition in low and middle income countries and recommended strengthening of food control systems as a necessary action to meet global nutrition goals [10, 11]. Quantitative assessments of how foods and foodborne disease hazards are related are the foundation for effective management of these diseases [12].

In 2015, the World Health Organization (WHO) Foodborne Disease Burden Epidemiology Reference Group (FERG) published the world’s first estimates of the global and regional incidence and burden of foodborne disease (FBD). This research estimated that in 2010, 31 major foodborne hazards resulted in over 600 million illnesses and 420,000 deaths worldwide in 2010 [13]. This represents an underestimation of the total incidence of foodborne disease, in particular because chemical hazards could not be fully addressed.

Estimates of the incidence of cases or mortality do not give a comprehensive picture of the impact of disease because disease severity and health outcomes vary across diseases. In the 1990s a comprehensive metric, the Disability-Adjusted Life Year (DALY) was developed as a means of comparing disease burden in global burden of disease studies [14]. DALYs are calculated by adding the number of years of life lost due to premature mortality (YLLs) and the number of years lived with disability (YLDs) from a disease or condition, adjusted for the severity of the disease [15]. One DALY is equivalent to one year of healthy life lost. The global burden of FBD caused by the 31 hazards (including sequelae) in 2010 was 33 million DALYs [13].

In a separate study, FERG researchers also studied the relationship between these illnesses and food exposures. An emerging area of research, food source attribution research, focuses on portioning FBD incidence among possible food exposure routes [8, 16]. The FERG’s Source Attribution Task Force (SATF) estimated the proportion of disease caused by 11 foodborne hazards that was the result of consumption of various foods, including beef, small ruminants’ meat, dairy products, pork, poultry meat, eggs, vegetables, fruits and nuts, grains and beans, oils and sugar, finfish, shellfish, and seaweed for 14 subregions [9]. The FERG results have shown that some pathogens of most concern, such as non-typhoidal *Salmonella enterica* (NTS), *Campylobacter* spp., Shiga-toxin producing *Escherichia coli* (STEC), *Toxoplasma gondii* and *Taenia solium* have livestock as their only or an important reservoir and can be transmitted directly or indirectly to humans.

In this study, we combine FERG results on FBD burden with FERG results on food source attribution to evaluate the role of ASF in causing foodborne disease around the world. We estimate the disease burden attributable to ASF globally and in 14 subregions. To provide context, we also examine the relationship between FBD burden from ASF and regional income. Income is an important driver behind both consumption of ASF and behind investments in strong food safety systems [2, 17, 18]. Our results should be useful in informing risk assessment, priority setting, development of targeted intervention strategies, and food safety management in general.

## Materials and methods

We combined three sets of estimates to estimate the disease burden of ASF food groups for different hazards globally and in different global subregions. First, we started with estimates of the disease burden for each of 31 potentially foodborne hazards using FERG estimates of disease burden in 2010. These estimates are expressed as DALYs per 100,000 population and were available for 194 countries in 14 subregions [13]. Second, we used FERG estimates attributing total disease for each of these 31 hazards to food versus non-food exposure routes [19]. Finally, we used FERG food source estimates to represent the proportion of the hazard-specific foodborne disease burden that was attributable to the consumption of specific types of food, including ASFs, for each of the 14 subregions [9]. The reference year for all of the FERG estimates is 2010.

### Data

Following FERG, we develop disease burden and source attribution estimates for 14 world subregions. These 14 subregions were developed by WHO for work on global burden of disease research [20]. The subregions are defined on the basis of the six official WHO regions, including the African Region (AFR), the Region of the Americas (AMR), the Eastern Mediterranean Region (EMR), the European Region (EUR), the South-East Asia Region (SEAR), and the Western Pacific Region (WPR). These regions were further subdivided into 14 subregions based on the mortality of children (under 5 years of age) and “adults” (≥ 5 years of age). The subregions were stratified on a scale ranging from A to E, with A having the lowest mortality rates and E the highest. To be more specific, stratum A indicates very low child and adult mortality, stratum B indicates low child mortality and very low adult mortality, stratum C represents low child mortality and high adult mortality, stratum D means high child and adult mortality, and stratum E represents high child mortality and very high adult mortality [20]. This subregional classification reflects overall development levels and water and sanitation conditions, factors that also influence food handling and storage conditions. The countries included in each of the 14 subregions are provided in [13] and are reproduced in S1 Table.

Among the 31 hazards in the FERG estimates of FBD burden, 13 were considered to be associated with ASF and thus included in this study. Of these, seven hazards were considered to be transmitted exclusively by one ASF group [19]. *Mycobacterium bovis* was attributed only to dairy products, *T*. *solium* and *Trichinella* spp. only to pork, *Paragonimus* spp. only to shellfish (including crustaceans), and foodborne trematodes (i.e. intestinal flukes, *Clonorchis sinensis*, and *Opisthorchis* spp.) only to finfish. The remaining six pathogens, including *Campylobacter* spp., STEC, NTS, *Cryptosporidium* spp., *Brucella* spp., and *T*. *gondii* were assessed to be transmitted by more than one food group, and a structured expert elicitation study was performed to estimate the proportion transmitted by each putative food group [9]. Table 1 shows the ASF food groups involved in transmission of these pathogens. The FERG food source attribution study included eight main groups of ASF: beef, pork, poultry, small ruminants’ meat, dairy, eggs, finfish and shellfish [9]. It ignored less frequently consumed meats, such as horse meat, bear meat and dog meat. All groups of ASFs were associated with multiple hazards. In total, there were 35 combinations of hazards and ASF food groups.

**Table 1:** Animal source foods involved in exposure to 13 different pathogens [9, 19].

	Animal source foods							
Hazards	Beef	Pork	Poultry	Small ruminant meat*	Dairy	Eggs	Finfish	Shellfish^1^
*Campylobacter* spp.	×	×	×	×	×			
Shiga-toxin producing *Escherichia coli*	×	×		×	×			
Non-typhoidal *Salmonella enterica*	×	×	×	×	×	×	×	×
*Cryptosporidium* spp.					×			
*Brucella* spp.	×	×		×	×			
*Mycobacterium bovis*					u			
*Toxoplasma gondii*	×	×	×	×	×	×		
*Taenia solium*		u						
*Trichinella* spp.		u^2^						
*Clonorchis sinensis*							u	
Intestinal flukes							u^3^	
*Opisthorchis* spp.							u	
*Paragonimus* spp.								u

×: hazard transmitted by this and other food groups (animal source or not), included in expert elicitation.

u: hazard transmitted only by one food group.

^1^ Including crustaceans.

^2^ Including wild boar meat. We neglect the small proportion of cases associated with meat from horses, bears and other animals.

^3^ Includes selected species of the families *Echinostomatidae*, *Gymnophallidae*, *Heterophyidae*, *Nanophyetidae*, *Neodiplostomidae* and *Plagiorchiidae*. We neglect transmission by other foods, such as shellfish, frogs, snails and snakes.

*Small ruminant meat primarily includes goat, sheep and lamb meat

### Data analysis

**Fig 1:**
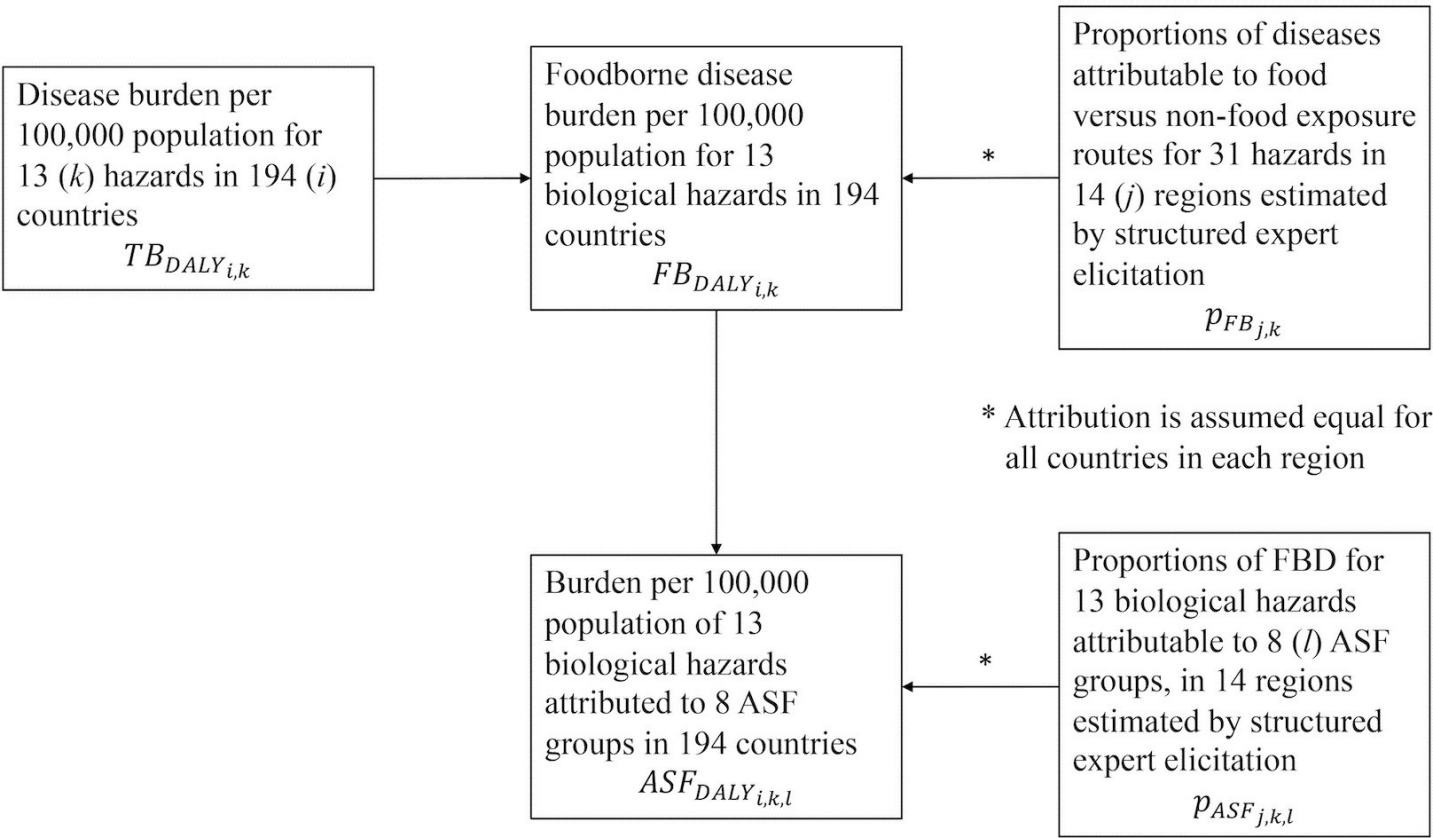
Flowchart of calculating ASF disease burden per 100,000 population.

Fig 1 shows the flowchart of calculating ASF disease burden per 100,000 population for 13 hazards and 8 ASFs in 14 subregions based on three previous FERG studies [9, 13, 19].

First, ${FB}_{{DALY}_{i,k}}$, the burden of FBD per 100,000 population in country *i* (*i* = 1, 2, …, 194) due to hazard *k* (*k* = 1, 2, …, 13), was calculated from the FERG estimates by multiplying ${TB}_{{DALY}_{i,k}}$, the total disease burden by each hazard in each country (16) with $p_{{FB}_{j,k}}$, the proportion of diseases attributable to food exposure routes estimated by structured expert elicitation [19]. Note that while FERG estimates are presented at subregional level, the calculations of disease burden were actually done at country level. The proportion of foodborne disease attribution was available for each hazard in *j* = 14 subregions and assumed to be the same in each country in any subregion.

Second, ${FB}_{{DALY}_{i,k}}$, was multiplied with $p_{{ASF}_{j,k,l}}$, the proportion of FBD burden attributed to specific animal products *l* (*l* = beef, small ruminant meat, dairy, pork, poultry meat, eggs, finfish, shellfish) by subregion *j* for each hazard *k* again assuming that this proportion was constant for each country in any subregion [9]. This provides an estimate of ${{ASF}_{DALY}}_{i,k,l}$, the burden per 100,000 of each individual hazard in each country attributed to each individual ASF group.

Statistical uncertainties in burden estimates were propagated using 10,000 Monte Carlo simulations in the statistical programming environment **R** version 3.4.4 [21] to calculate estimates of the distribution of incidence, mortality, and DALYs. The distributions were then summarized by their median and a 95% uncertainty interval (UI) defined as the distribution’s 2.5th and 97.5th percentile.

Results are reported below as the disease burden (DALYs) per 100,000 population due to consumption of ASF food groups for different combinations of the 8 ASF products, 13 hazards, and 14 subregions, as well as globally by aggregating over different indices of ${{ASF}_{DALY}}_{i,k,l}$.

### Availability of data

Data availability is restricted by the World Health Organization because results at country level have not been cleared with Member States. Data is available from the authors after obtaining permission from the World Health Organization Department of Food Safety and Zoonoses (foodsafety@who.int).

## Results

Globally, the median burden of all hazards due to ASF consumption was 168 (95% uncertainty interval (UI) 137–219) DALYs per 100,000 population (Table 2). This represents approximately 35% of the burden of foodborne disease due to all foods (477 DALYs (95% UI 361–673) per 100,000 population) [13]. Three pathogens, NTS, *T*. *solium*, and *Campylobacter* spp. caused approximately 70% of this burden (49 (95% UI 30–76), 41 (95% UI 31–52), and 27 (95% UI 19–40) DALYs per 100,000 population respectively), while *Cryptosporidium* spp., STEC, and *Trichinella* spp. caused the lowest ASF disease burden (0.3 (95% UI 0.1–2), 0.1(95% UI 0.1–0.4), and 0.01 (95% UI 0.004–0.01) DALYs per 100,000 population, respectively).

The median ASF burden in the African subregions AFR D and E was 580 (95% UI 314–879) and 459 (95% UI 294–625) DALYs per 100,000 population, respectively, a burden that is remarkably higher than those in the “A” subregions including AMR A, WPR A, and EUR A (ranging between 21 and 25 DALYs per 100,000 population). The median ASF burden in other regions was intermediate, and as the 95% uncertainty intervals are relatively wide, no further conclusions on relative differences can be drawn.

**Table 2:** Burden (Disability-Adjusted Life Years per 100,000 population) due to consumption of animal source foods, 2010 (median, 95% uncertainty interval).

Subregion	*Camp*.	STEC	NTS	*Cryp*.	*Bruc*.	*Toxo*.	*Myco*.	*T*. *sol*.	*Tric*.	*Clon*.	Fluke	*Opis*.	*Para*.	All hazards
Global	27 (19–40)	0.1 (0.1–0.4)	49 (30–76)	0.3 (0.1–2)	2 (0.6–41)	9 (6–14)	9 (7–12)	41 (31–52)	0.01 (0.004–0.01)	8 (6–9)	2 (2–3)	3 (2–3)	15 (11–21)	168 (137–219)
AFR D	62 (28–106)	0.01 (0–0.02)	286 (79–529)	0.4 (0–8)	2 (0.2–51)	16 (7–33)	25 (15–39)	170 (110–283)	0.001 (0.0005–0.002)	0 (0–0)	0.01 (0–0.04)	0 (0–0)	0.03 (0.01–0.08)	580 (314–879)
AFR E	62 (28–106)	0.07 (0.02–0.2)	163 (37–293)	0.4 (0–10)	0.3 (0.01–17)	16 (7–30)	34 (21–48)	176 (134–229)	0.001 (0.0005–0.002)	0 (0–0)	0 (0–0)	0 (0–0)	0.01 (0–0.02)	459 (294–625)
AMR A	8 (4–13)	0.1 (0.03–0.3)	8 (3–13)	0.01 (0–0.1)	0.07 (0.02–0.6)	4 (2–7)	0.03 (0.01–0.06)	0.4 (0.3–0.6)	0.009 (0.005–0.01)	0 (0–0)	0.1 (0.04–0.5)	0 (0–0)	0.04 (0–0.6)	21 (12–30)
AMR B	13 (7–21)	0.3 (0.07–1)	9 (2–16)	0.05 (0–0.5)	1 (0.3–15)	15 (7–26)	0.4 (0.2–0.8)	25 (19–32)	0.009 (0.005–0.01)	0 (0–0)	0.06 (0.02–0.2)	0 (0–0)	0.04 (0.01–0.1)	65 (48–88)
AMR D	14 (7–24)	0.4 (0.1–0.9)	12 (3–22)	0.09 (0–0.7)	2 (0.2–35)	20 (7–67)	2 (0.8–4)	69 (51–91)	0.009 (0.005–0.01)	0 (0–0)	0 (0–0)	0 (0–0)	53 (38–73)	176 (141–254)
EMR B	50 (23–86)	0.2 (0.07–0.3)	42 (15–71)	0.1 (0–4)	23 (3–81)	15 (7–27)	1 (0.5–3)	0 (0–0)	0.0001 (0–0.0003)	0 (0–0)	0.06 (0.02–0.2)	0 (0–0)	0 (0–0)	136 (78–215)
EMR D	87 (47–131)	0.2 (0.08–0.4)	57 (21–98)	0.09 (0–4)	4 (0.6–64)	13 (6–23)	13 (6–25)	0 (0–0)	0.0001 (0–0.0003)	0 (0–0)	0.08 (0.03–0.2)	0 (0–0)	0.02 (0.01–0.07)	180 (104–277)
EUR A	9 (5–13)	0.5 (0.2–1)	11 (6–17)	0 (0–0.1)	0.3 (0.07–1)	4 (2–7)	0.08 (0.06–0.1)	0 (0–0)	0.04 (0.02–0.07)	0 (0–0)	0.03 (0.01–0.09)	0.07 (0.02–0.3)	0 (0–0)	26 (17–34)
EUR B	7 (3–11)	0.06 (0.01–0.2)	10 (5–18)	0 (0–0.2)	4 (0.6–33)	8 (3–18)	0.6 (0.5–1)	0 (0–0)	0.04 (0.02–0.07)	0 (0–0)	0.05 (0.02–0.2)	0.05 (0.01–0.3)	0 (0–0)	31 (19–67)
EUR C	7 (3–11)	0.1 (0.02–0.3)	9 (4–16)	0.01 (0–0.2)	0.8 (0.07–6)	7 (3–13)	3 (2–5)	1 (0.6–2)	0.04 (0.02–0.07)	0.04 (0.03–0.04)	0.09 (0.03–0.2)	0.9 (0.6–1)	0.03 (0.01–0.1)	29 (20–43)
SEAR B	32 (12–78)	0.1 (0.01–0.8)	47 (16–127)	0.1 (0–4)	0.8 (0–108)	8 (4–17)	11 (4–27)	3 (2–5)	0.0007 (0.0002–0.001)	0.01 (0–0.04)	0.2 (0.1–0.5)	40 (32–50)	0.05 (0.01–0.5)	156 (100–291)
SEAR D	27 (2–73)	0.1 (0–0.8)	45 (0–130)	0.1 (0–5)	0.7 (0–87)	6 (1–14)	14 (6–27)	45 (33–60)	0.0007 (0.0002–0.001)	0.04 (0.01–0.2)	0.1 (0.03–0.4)	0.4 (0.1–2)	0.06 (0.02–0.2)	156 (100–291)
WPR A	8 (5–13)	0.3 (0.1–1)	7 (4–13)	0 (0–0.1)	0.6 (0.02–123)	4 (2–7)	0.1 (0.08–0.2)	0 (0–0)	0.0004 (0.001–0.007)	0.05 (0.01–0.2)	1.4 (0.9–2)	0 (0–0)	0.05 (0.02–0.2)	25 (16–143)
WPR B	8 (3–15)	0.01 (0–0.03)	7 (3–13)	0.01 (0–0.4)	0.6 (0.09–9)	7 (3–11)	3 (1–5)	27 (20–35)	0.004 (0.001–0.007)	31 (26–38)	9 (7–11)	3 (2–4)	60 (43–83)	158 (132–189)

Abbreviations: *Camp*.: *Campylobacter* spp.; STEC: Shiga-toxin producing *Escherichia coli*; NTS: Non-typhoidal *Salmonella enterica*; *Cryp*.: *Cryptosporidium* spp.; *Bruc*.: *Brucella* spp.; *Toxo*.: *Toxoplasma gondii*; *Myco*.: *Mycobacterium bovis*; *T*. *sol*.: *Taenia solium*; *Tric*.: *Trichinella* spp.; *Clon*.: *Clonorchis sinensis*; Fluke: Intestinal flukes; Opis.: *Opisthorchis* spp.; *Para*.: *Paragonimus* spp.

**Fig 2:**
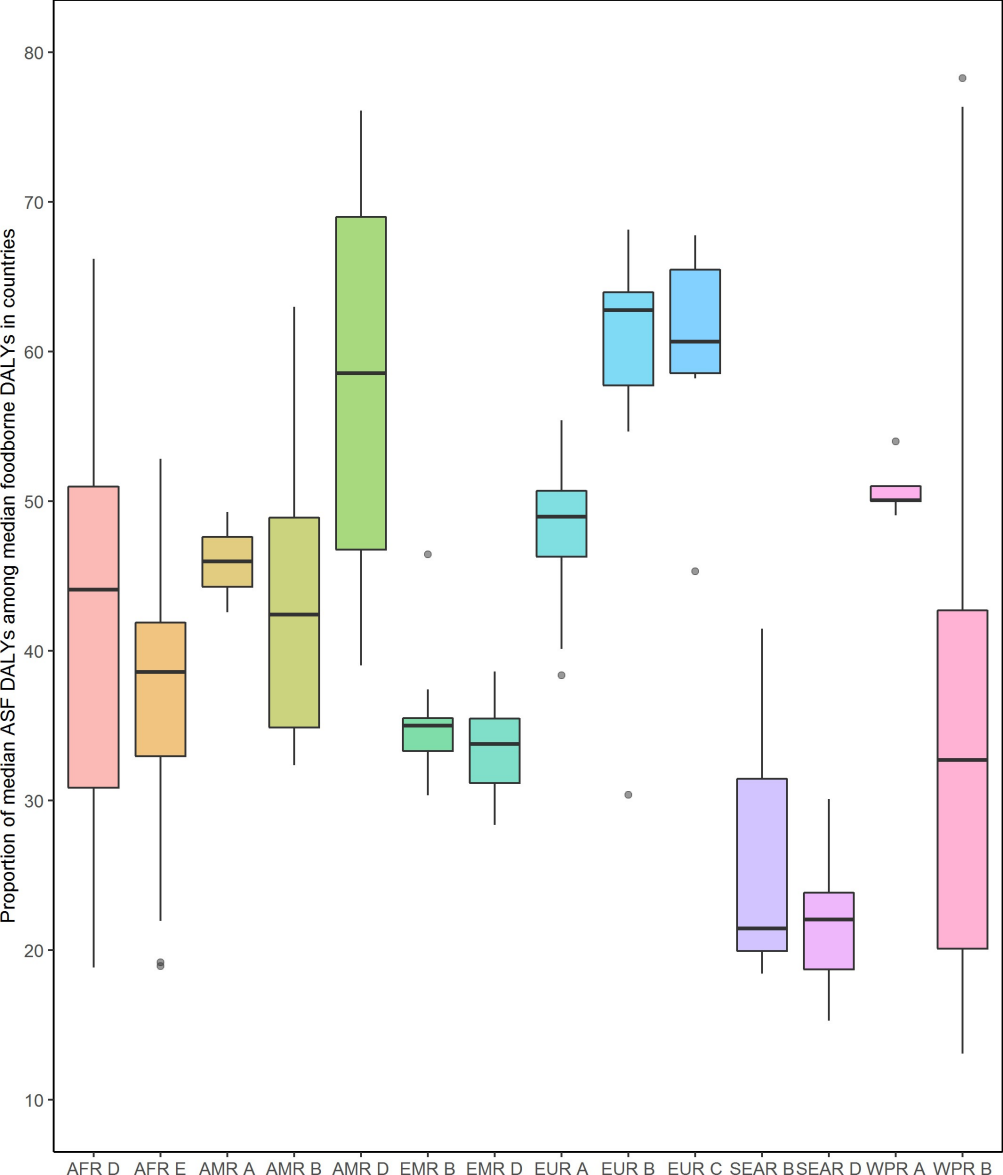
The proportion of median Disability-Adjusted Life Years from animal source foods among median foodborne Disability-Adjusted Life Years is highly variable between countries in each subregion and between subregions (see text for abbreviations).

There were large differences in the proportion of foodborne disease burden (median of DALYs per 100,000 populations) attributed to ASF in the 14 subregions and between countries in each subregion (Fig 2). The highest median percentage of foodborne disease burden attributed to ASF was observed in EUR B, EUR C, and AMR D, and the lowest in SEAR B, SEAR D, and WPR B. There were also considerable variations of the percentage among countries in several subregions, such as AFR D, AMR D, SEAR B, and WPR B, which had especially wide spans of country-specific ASF percentages. Subregions with relatively small variations of the ASF percentages included AMR A (with only three countries), EMR B, and WPR A.

Table 3 presents the proportion of FBD burden from the 13 pathogens included in this study that was attributable to ASF by different hazards and in different subregions. Approximately 90% of the FBD burden of *Campylobacter* spp. was attributed to ASF in all regions except in EMR B, where a relatively low proportion of 66% was estimated, while 33% of the burden was attributed to vegetables. The ASF-attributable proportion of *Brucella* spp. in all subregions was above 95%, while those of STEC, NTS and *T*. *gondii* were approximately 70–80%. The ASF-attributable proportions of *Cryptosporidium* spp. were less than 10% in all subregions, which was the lowest among all hazards. *M*. *bovis*, *T*. *solium*, *Trichinella* spp., intestinal flukes, *C*. *sinensis*, *Opisthorchis* spp. and *Paragonimus* spp. were all attributed only to one ASF food product, as discussed earlier.

**Table 3:** Proportion (%) of foodborne disease burden attributable to animal source foods for different hazards, 2010 (median, 95% uncertainty interval).

Subregion	*Campylobacter* spp.	STEC	Non-typhoidal *Salmonella enterica*	*Cryptosporidium* spp.	*Brucella* spp.	*Toxoplasma gondii*
AFR D	91% (72%-98%)	83% (58–96%)	84% (63–94%)	3% (0–42%)	95% (89–100%)	80% (50–95%)
AFR E	91% (71–98%)	85% (59–96%)	84% (63–94%)	4% (0–46%)	95% (89–100%)	79% (55–95%)
AMR A	90% (70–99%)	77% (52–95%)	83% (62–94%)	5% (0–29%)	99% (92–100%)	80% (52–96%)
AMR B	92% (73–98%)	81% (57–94%)	80% (58–93%)	8% (0–35%)	98% (89–100%)	77% (49–95%)
AMR D	92% (73–98%)	81% (56–94%)	81% (59–92%)	8% (0–36%)	95% (87–100%)	78% (48%-97%)
EMR B	66% (41–96%)	84% (57–95%)	84% (65–93%)	4% (0–49%)	99% (90–100%)	74% (50–95%)
EMR D	92% (72–99%)	84% (56–95%)	84% (64–94%)	3% (0–49%)	95% (89–100%)	72% (47–93%)
EUR A	90% (70–98%)	78% (54–95%)	89% (64–98%)	4% (0–40%)	99% (96–100%)	75% (48–93%)
EUR B	86% (65–98%)	82% (54–96%)	84% (59–95%)	4% (0–44%)	98% (94–100%)	77% (51–93%)
EUR C	86% (64–98%)	82% (54–96%)	84% (60–95%)	5% (0–49%)	95% (89–100%)	74% (47–93%)
SEAR B	89% (67–98%)	73% (40–93%)	79% (53–93%)	3% (0–38%)	98% (92–100%)	74% (52–94%)
SEAR D	86% (54–98%)	73% (40–93%)	77% (51–92%)	2% (0–40%)	95% (89–100%)	70% (43–91%)
WPR A	91% (71–99%)	83% (54–97%)	85% (62–96%)	2% (0–33%)	99% (94–100%)	81% (55–95%)
WPR B	89% (66–98%)	74% (43–94%)	81% (55–94%)	2% (0–44%)	98% (94–100%)	80% (57–96%)

Other hazards were exclusively attributed to one specific ASF and thus have a attribution proportion of 100%, i.e. *Mycobacterium bovis* to dairy products, *Taenia solium* and *Trichinella* spp. to pork, *Paragonimus* spp. to shellfish, and foodborne trematodes (*Clonorchis sinensis*, Intestinal flukes, and *Opisthorchis* spp.) to finfish.

**Fig 3:**
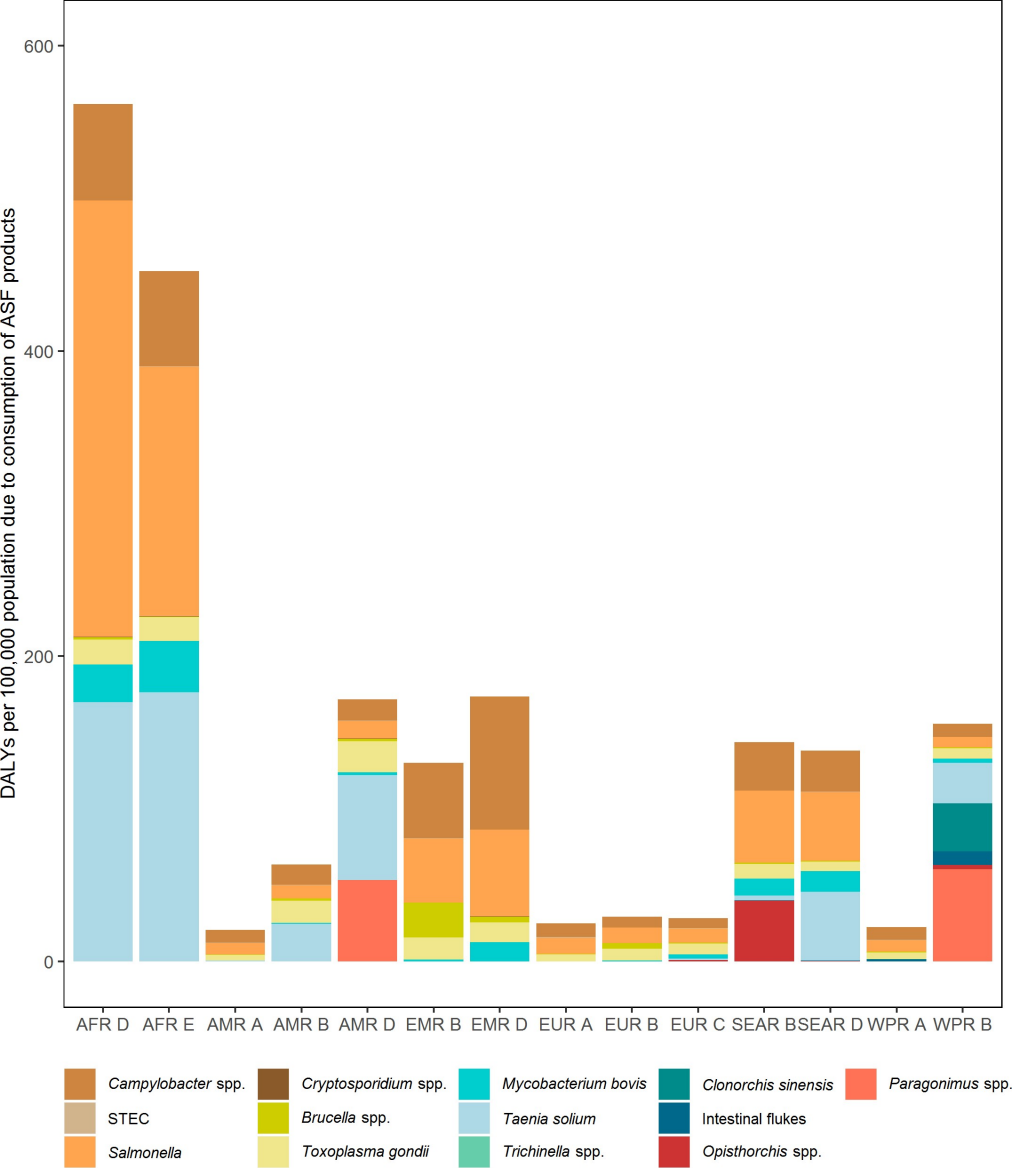
The burden due to consumption of animal source foods for 13 hazards is highest in Africa. Different pathogens contribute most to this burden in different subregions. For abbreviations see Fig 2.

The contribution of individual hazards to the burden of ASF per 100,000 population differed markedly between subregions (Fig 3). The two African subregions had much higher ASF burden than in other subregions, and NTS and *T*. *solium* dominated the burden in these two subregions. In the American subregions, NTS and *Campylobacter* spp. accounted for nearly 75% of the total burden in AMR A, while *T*. *solium* contributed considerable burden in both AMR B (approximately 40%) and AMR D (approximately 40%) subregions. In addition to the AMR A subregion, *Salmonella* and *Campylobacter* spp. together also contributed most of the ASF burden in the EMR, the EUR, the SEAR, and the WPR A (sub)regions. The disease burden for some foodborne trematodes almost exclusively occurred in certain subregions, for example, *Clonorchis* spp. only occurred in the WPR B subregion and *Opisthorchis* spp. only in the SEAR B subregion.

**Fig 4:**
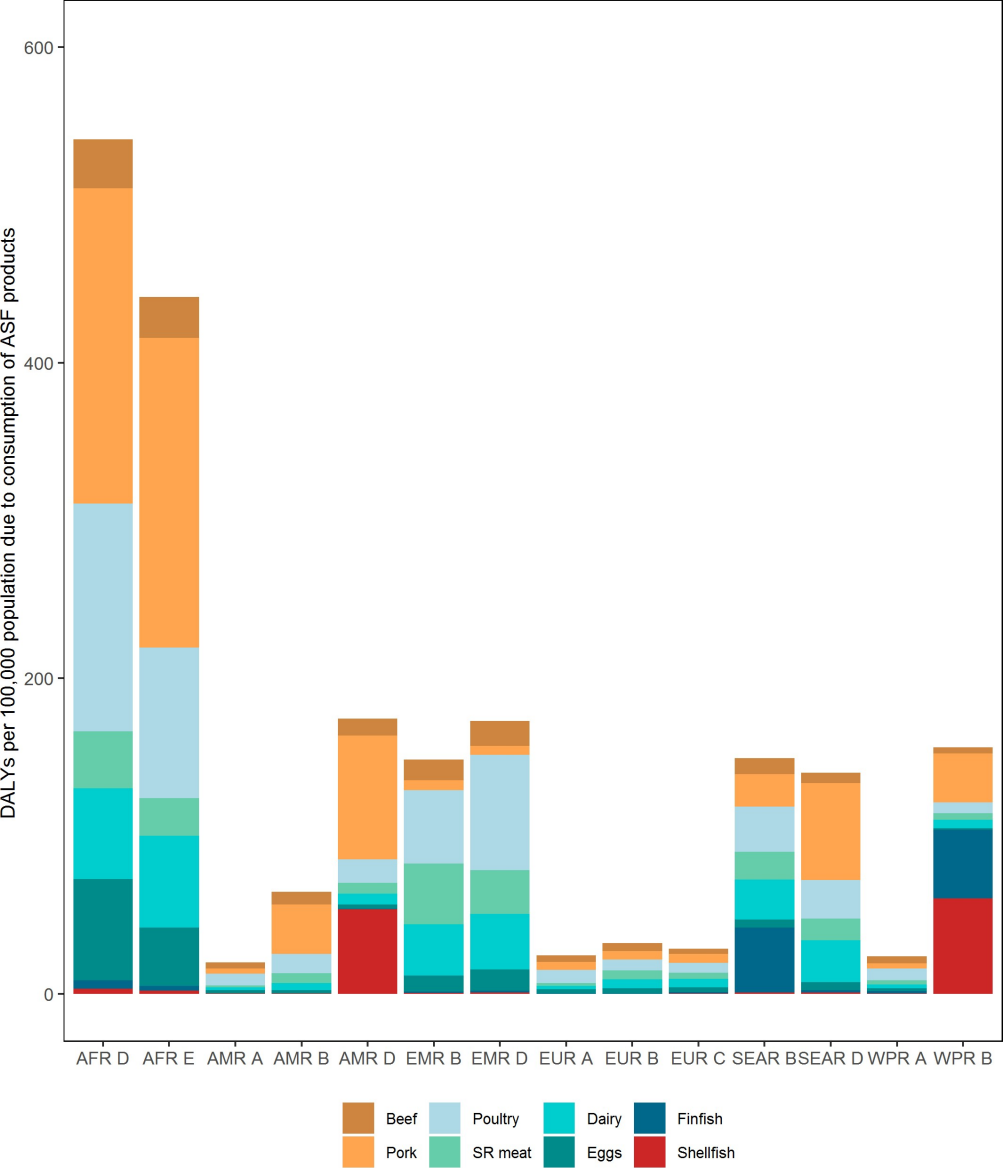
Different food groups contribute differently to the burden due to consumption of animal source foods in each subregion. SR meat–small ruminant meat. For other abbreviations see Fig 2.

The ASF disease burden was mainly attributed to some dominant ASF food groups in certain subregions (Fig 4 and S2–S9 Tables). For example, the disease burden due to pork consumption was very high in AFR D (200 (95% UI 129–325) DALYs per 100,000 population), AFR E (196 DALYs (95% UI 148–261) per 100,000 population), AMR B (32 DALYs (95% UI 24–41) per 100,000 population), AMR D (78 (95% UI 60–106) DALYs per 100,000 population) and SEAR D (62 (95% UI 42–99) DALYs per 100,000 population) (S3 Table). ASF attributable to pork was low in EMR, particularly when adjusted for pork consumption data. Poultry was a major contributor to the disease burden in EMR B (46 (95% UI 20–82) DALYs per 100,000 population) and EMR D (73 (95% UI 31–124) DALYs per 100,000 population) and also contributed considerably to the disease burden in AFR D (144 (95% UI 37–292) DALYs per 100,000 population) and AFR E (96 (95% UI 27–182) DALYs per 100,000 population) (S4 Table). Finfish and shellfish contributed substantial disease burden to SEAR B (41 (95% UI 33–52) DALYs per 100,000 population) (S8 Table) and WPR B (61 (95% UI 42–83) DALYs per 100,000 population) (S9 Table), respectively. Dairy caused similar disease burden in both AFR D (58 (95% UI 27–143) DALYs per 100,000 population) and AFR E (58 (95% UI 35–99) DALYs per 100,000 population) (S6 Table) and eggs led to 64 (95% UI 0.01–176) DALYs per 100,000 population and 37 (95% UI 0.02–99) DALYs per 100,000 population in AFR D and AFR E (S7 Table), respectively. Beef and small ruminant meat caused a relatively minor disease burden in all subregions (S2 and S5 Tables).

**Fig 5:**
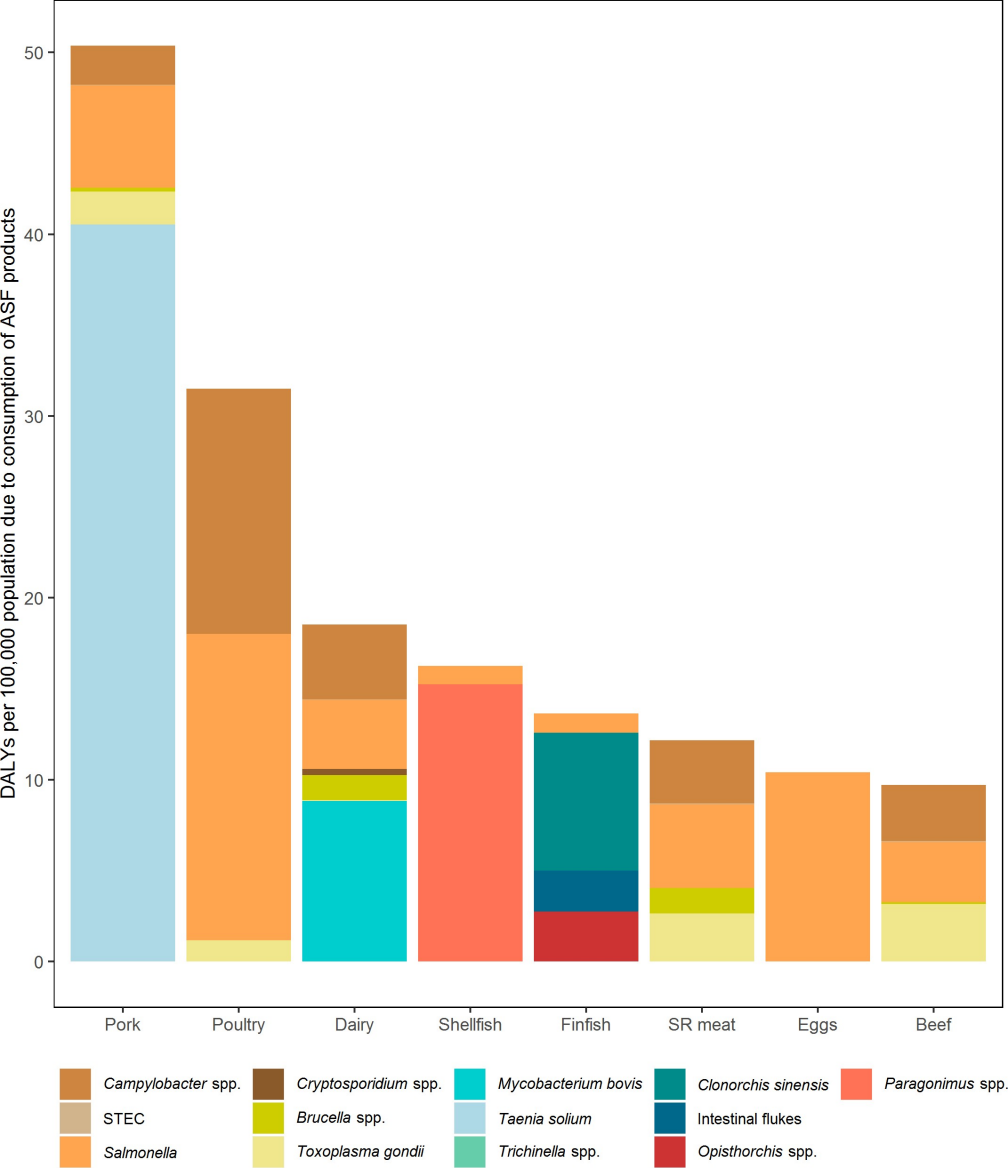
Different pathogens contribute to the burden of different animal source food groups. STEC: Shiga-toxin producing *Escherichia coli*. For other abbreviations see Fig 2.

The median DALYs per 100,000 population were highest for consumption of pork (51), poultry (32) and dairy (20), followed by shellfish (16), finfish (14), small ruminant meat (13), eggs (10), and beef (10) (Fig 5). In addition, *T*. *solium* caused approximately 80% of the disease burden from pork. *Salmonella* and *Campylobacter* spp. dominated the disease burden from poultry (approximately 90%), *M*. *bovis* contributed most to the disease burden from dairy (approximately 50%), and *Paragonimus* spp. and *Clonorchis* spp. dominated the disease burden from shellfish (approximately 90%) and finfish (approximately 60%), respectively. Disease burden from eggs was almost exclusively associated with NTS, with a very small proportion from *T*. *gondii*. Furthermore, *Campylobacter* spp., *Salmonella*, and *T*. *gondii* contributed similar proportions of disease burden to beef and small ruminant meat.

The results presented so far represent risks associated direct exposure from ASF, and do not reflect indirect transmission of disease agents from livestock production systems. Table 4 breaks down the burden of disease of the six most important pathogens that have food animals as their main reservoir [6, 8]. Four of these were transmitted exclusively by food, but two (NTS and *Campylobacter* spp.) could also be transmitted by other pathways, such as waterborne transmission, direct animal contact, and human to human contact. Ultimately, the main reservoirs for NTS and for *Campylobacter* spp. are livestock [22, 23]. Hald et al. [19] provide global source attribution estimates for food and other possible exposure routes, including direct contact with livestock. Using these broader source attribution estimates, we found an additional burden of approximately 86 DALYs per 100,000, attributed to non-foodborne transmission of these two pathogens. Together, these estimates indicate that production and consumption of ASF imposes a burden of 254 DALYs per 100,000 rather than the 168 DALYs per 100,000 associated directly with food consumption.

**Table 4:** Exposure pathways for *Campylobacter* spp. and non-typhoidal *Salmonella enterica* associated with animal source foods, 2010.

Pathway	*Campylobacter* spp.	Non-typhoidal *Salmonella enterica*
All pathways^1^	54 (42–77)^2^	122 (92–167)
All food^3^	31 (22–46)	59 (36–91)
ASF	27 (19–40)	49 (30–76)
Beef	3 (1–6)	3 (1–8)
Pork	2 (0.8–6)	6 (2–12)
Poultry	13 (9–20)	17 (9–29)
SR meat	3 (2–7)	5 (1–13)
Dairy	4 (2–8)	4 (2–9)
Eggs		10 (5–19)
Finfish		1 (0.4–3)
Shellfish		1 (0.3–4)

^1^ Based on S18 Table in online supplementary information in Havelaar et al. [13].

^2^ Median global burden (Disability-Adjusted Life Years per 100,000) and 95% uncertainty interval.

^3^ Based on Havelaar et al. [13].

## Discussion

We present a study on the global disease burden associated with 13 pathogens with animal reservoirs in 2010, and showed that, approximately one-third of the FBD burden from these pathogens was associated with consuming ASF. The proportion of FBD from these pathogens that was associated with ASF was highly variable between subregions and countries. For several pathogens, including the pig tapeworm *T*. *solium*, trematodes, and *M*. *bovis*, this variation in FBD was related to the geographically restricted ranges of the organisms. Other pathogens, including NTS, *Campylobacter* spp. and *T*. *gondii*, have a global distribution and therefore affected FBD burden globally, in high-income countries as well low- and middle-income countries [13]. These differences need to be accounted for when using the current results for decision making, e.g. in ranking pathogen-specific foodborne disease risks.

We have no complete evidence on the contributions of other foods to the global burden of foodborne disease. Key pathogens contributing to this burden, such as *Salmonella* Typhi, enteropathogenic *Escherichia coli*, norovirus and enterotoxigenic *E*. *coli* and hepatitis A virus [13] all have exclusively or predominantly human reservoirs. These may be transmitted by foods such as vegetables and fruits that are contaminated at primary production or during harvest and retail, but may also be spread by cross-contamination on the kitchen environment, either at home or in professional kitchens.

The FERG estimates have some limitations. The first of these is due to data gaps, particularly in low-income countries where the burden is highest. We attempted to address these data gaps by imputation and using expert elicitation, which contributed to higher levels of overall uncertainty [15]. We were unable to include burden estimates of some foodborne pathogens where ASF are important. In particular, there were no attribution estimates for *Listeria monocytogenes*, because the pathogen is ubiquitous in the environment. Nor were there attribution estimates for transmission of viruses or *Vibrio* species through shellfish, but the burden associated with these foods was small compared to other food-hazard pairs and thus not included in the current study [13]. In addition, the expert elicitation suggested some burden due to pork transmitted pathogens in the EMR region. According to FAO STAT, the only countries in this region to have significant pork consumption are Cyprus and Lebanon, which is not surprising as the other countries in this region are overwhelmingly Muslim majority countries. Thus, an alternative scenario taking into account this pork consumption pattern is reported in S3 Table. Of 5 experts providing estimates for the proportion of salmonellosis attributable to pork, one expert indeed provided very low estimates (median proportion 0.0001%), whereas the median estimates of other experts ranged between 3 and 7%.

It is not yet possible to quantify the burden of malnutrition associated with ASF consumption. In 2016, there were an estimated 815 million chronically undernourished people in the world, primarily in Sub-Saharan Africa. Of these, 155 million children suffered from stunting, primarily in Sub-Saharan Africa and South-East Asia. Among the key determinants of stunting are compromised maternal health and nutrition before and during pregnancy and lactation, inadequate breastfeeding, poor feeding practices for infants and young children including unsafe foods, and unhealthy environments for children, including poor hygiene and sanitation [24]. Stunting mainly occurs in children under 2 years of age, is nearly irreversible and is associated with increased morbidity and mortality, reduced physical, neurodevelopmental and economic capacity and an elevated risk of metabolic disease into adulthood [25]. Diarrheal disease is a major risk factor for undernutrition in children under 5 years of age. Troeger et al. [26] concluded that each day of diarrhea was associated with significant decreases in height-for-age Z-score, weight-for-age Z-score and weight-for-height Z-score. According to these authors, the increased susceptibility to infectious diseases resulting from undernutrition (diarrhea, higher rates of respiratory infections, and measles), increased the burden of diarrheal diseases by 39.0% (95% UI 33.0–46.6) and were responsible for 55,778,000 DALYs (95% UI 49,125,400–62,396,200) among children younger than 5 years in 2016. Furthermore, chronic, asymptomatic gut infections by enteric pathogens are an important cause of environmental enteric dysfunction, one of the major contributing factors to malnutrition and stunting. *Campylobacter* spp. are among the most important pathogens associated with Environmental Enteric Dysfunction and stunting [27, 28]. Little is known about exposure pathways of young children in low- and middle-income countries, but it is likely that livestock are the main reservoirs of these bacteria, with multiple exposure pathways including foodborne transmission.

Nevertheless ASF are rich sources of macro- and micronutrients, many of them more bioavailable than in plant foods [1]. As discussed above, nutrition research shows that consumption of ASF improves maternal health, child growth and cognitive function, yet they are often lacking in the diets of children and pregnant and lactating women in low- and middle-income countries. As a result, there is increasing call for promoting consumption of ASFs in low- and middle-income countries particularly among children and pregnant and lactating women to address chronic undernutrition problems. Our results provide a very concrete demonstration of the Second International Conference on Nutrition 2014 recognition that food safety is a necessary enabling environment for improving nutrition in low income countries [10, 29, 30].

Control methods exist for many hazards in ASF. The burden of foodborne disease from ASF is very similar in in all high-income regions and in the EUR B and C subregions (Fig 2), suggesting that this is the limit of what currently available control methods can achieve. The considerably higher burden in low- to middle-income countries suggests that implementation of effective ASF safety systems is linked to economic development. This implies an optimistic message: when countries get richer, their food safety systems evolve and the greater effectiveness of these systems is able to not only keep up with the increasing consumption of ASF but even makes these foods safer to consume. A recent analysis by the World Bank, based on FERG data, suggests that the burden of ASF in Sub Saharan African countries with adequate levels of operational funding for veterinary services is 208 DALYs per 100,000 population, while it is 569 DALYs per 100,000 population in countries where such funding is inadequate [18]. For a country like Nigeria, inadequate funding of veterinary services would translate to annual production losses of US$ 1.3 billion. These authors conclude that “these results provide a compelling case that moderate levels of investment in enhancing food safety management capacity—and specifically for animal-based FBD—can have significant public health and economic benefits”. A key challenge is to adopt approaches that have proven successful in high-income countries in an economically and culturally acceptable way to low- and middle-income countries. Furthermore, many interventions to improve safety of ASF require multisector approaches, and may not be effective in isolation, consistent with calls for One Health initiatives, which address the linkages between livestock and human health.

## Supporting information

S1 Table World Health Organization (WHO) Member States by subregion. (DOCX)

S2 Table Burden (Disability-Adjusted Life Years per 100,000 population) due to consumption of beef, 2010 (median, 95% uncertainty interval). (DOCX)

S3 Table Burden (Disability-Adjusted Life Years per 100,000 population) due to consumption of pork, 2010 (median, 95% uncertainty interval). (DOCX)

S4 Table Burden (Disability-Adjusted Life Years per 100,000 population) due to consumption of poultry, 2010 (median, 95% uncertainty interval). (DOCX)

S5 Table Burden (Disability-Adjusted Life Years per 100,000 population) due to consumption of small ruminant meats, 2010 (median, 95% uncertainty interval). (DOCX)

S6 Table Burden (Disability-Adjusted Life Years per 100,000 population) due to consumption of dairy, 2010 (median, 95% uncertainty interval). (DOCX)

S7 Table Burden (Disability-Adjusted Life Years per 100,000 population) due to consumption of eggs, 2010 (median, 95% uncertainty interval). (DOCX)

S8 Table Burden (Disability-Adjusted Life Years per 100,000 population) due to consumption of finfish, 2010 (median, 95% uncertainty interval). (DOCX)

S9 Table Burden (Disability-Adjusted Life Years per 100,000 population) due to consumption of shellfish, 2010 (median, 95% uncertainty interval). (DOCX)
