# Supplementary material for: Global disease burden of pathogens in animal source foods, 2010
Source: PLoS One. 2019 Jun 6;14(6):e0216545. doi: 10.1371/journal.pone.0216545 (PMC6553721; doi:10.1371/journal.pone.0216545)
Supplement: S1 Table — (DOCX) [file pone.0216545.s001.docx]

S1 Table. World Health Organization (WHO) Member States by subregion (reproduced from [1])

| Subregions^1^ | WHO member states |
| --- | --- |
| AFR D | Algeria; Angola; Benin; Burkina Faso; Cameroon; Cape Verde; Chad; Comoros; Equatorial Guinea; Gabon; Gambia; Ghana; Guinea; Guinea-Bissau; Liberia; Madagascar; Mali; Mauritania; Mauritius; Niger; Nigeria; Sao Tome and Principe; Senegal; Seychelles; Sierra Leone; Togo. |
| AFR E | Botswana; Burundi; Central African Republic; Congo; Côte d'Ivoire; Democratic Republic of the Congo; Eritrea; Ethiopia; Kenya; Lesotho; Malawi; Mozambique; Namibia; Rwanda; South Africa; Swaziland; Uganda; United Republic of Tanzania; Zambia; Zimbabwe. |
| AMR A | Canada; Cuba; United States of America. |
| AMR B | Antigua and Barbuda; Argentina; Bahamas; Barbados; Belize; Brazil; Chile; Colombia; Costa Rica; Dominica; Dominican Republic; El Salvador; Grenada; Guyana; Honduras; Jamaica; Mexico; Panama; Paraguay; Saint Kitts and Nevis; Saint Lucia; Saint Vincent and the Grenadines; Suriname; Trinidad and Tobago; Uruguay; Venezuela (Bolivarian Republic of). |
| AMR D | Bolivia (Plurinational State of); Ecuador; Guatemala; Haiti; Nicaragua; Peru. |
| EMR B | Bahrain; Iran (Islamic Republic of); Jordan; Kuwait; Lebanon; Libyan Arab Jamahiriya; Oman; Qatar; Saudi Arabia; Syrian Arab Republic; Tunisia; United Arab Emirates. |
| EMR D | Afghanistan; Djibouti; Egypt; Iraq; Morocco; Pakistan; Somalia; South Sudan^2^; Sudan; Yemen. |
| EUR A | Andorra; Austria; Belgium; Croatia; Cyprus; Czech Republic; Denmark; Finland; France; Germany; Greece; Iceland; Ireland; Israel; Italy; Luxembourg; Malta; Monaco; Netherlands; Norway; Portugal; San Marino; Slovenia; Spain; Sweden; Switzerland; United Kingdom. |
| EUR B | Albania; Armenia; Azerbaijan; Bosnia and Herzegovina; Bulgaria; Georgia; Kyrgyzstan; Montenegro; Poland; Romania; Serbia; Slovakia; Tajikistan; The Former Yugoslav Republic of Macedonia; Turkey; Turkmenistan; Uzbekistan. |
| EUR C | Belarus; Estonia; Hungary; Kazakhstan; Latvia; Lithuania; Republic of Moldova; Russian Federation; Ukraine. |
| SEAR B | Indonesia; Sri Lanka; Thailand. |
| SEAR D | Bangladesh; Bhutan; Democratic People's Republic of Korea; India; Maldives; Myanmar; Nepal; Timor-Leste. |
| WPR A | Australia; Brunei Darussalam; Japan; New Zealand; Singapore. |
| WPR B | Cambodia; China; Cook Islands; Fiji; Kiribati; Lao People's Democratic Republic; Malaysia; Marshall Islands; Micronesia (Federated States of); Mongolia; Nauru; Niue; Palau; Papua New Guinea; Philippines; Republic of Korea; Samoa; Solomon Islands; Tonga; Tuvalu; Vanuatu; Viet Nam. |

^1^ The subregions are defined on the basis of child and adult mortality as described by Ezzati et al. [2] Stratum A: very low child and adult mortality, Stratum B: low child mortality and very low adult mortality, Stratum C: low child mortality and high adult mortality, Stratum D: high child and adult mortality, and Stratum E: high child mortality and very high adult mortality. The use of the term ‘subregion’ here and throughout the text does not identify an official grouping of WHO Member States, and the “subregions” are not related to the six official WHO regions.
AFR = African Region; AMR = Region of the Americas; EMR = Eastern Mediterranean Region; EUR = European Region; SEAR = South-East Asia Region; WPR = Western Pacific Region.

^2^ South Sudan was reassigned to the WHO African Region in May 2013. As this study relates to time periods prior to this date, estimates for South Sudan were included in the WHO Eastern Mediterranean Region.

1. Havelaar AH, Kirk MD, Torgerson PR, Gibb HJ, Hald T, Lake RJ, et al. World Health Organization Global Estimates and Regional Comparisons of the Burden of Foodborne Disease in 2010. PLoS Med. 2015;12(12):e1001923. doi: 10.1371/journal.pmed.1001923. PubMed PMID: 26633896; PubMed Central PMCID: PMCPMC4668832.

2. Ezzati M, Lopez AD, Rodgers A, Vander Hoorn S, Murray CJ, Comparative Risk Assessment Collaborating Group. Selected major risk factors and global and regional burden of disease. Lancet. 2002;360(9343):1347-60. doi: 10.1016/S0140-6736(02)11403-6. PubMed PMID: 12423980.
