# Supplementary material for: Global disease burden of pathogens in animal source foods, 2010
Source: PLoS One. 2019 Jun 6;14(6):e0216545. doi: 10.1371/journal.pone.0216545 (PMC6553721; doi:10.1371/journal.pone.0216545)
Supplement: S2 Table — (DOCX) [file pone.0216545.s002.docx]

S2 Table. Burden (Disability-Adjusted Life Years per 100,000 population) due to consumption of beef, 2010 (median, 95% uncertainty interval)

|  | *Brucella* spp. | *Campylobacter* spp. | NTS^1^ | STEC^2^ | *Toxoplasma gondii* | All hazards |
| --- | --- | --- | --- | --- | --- | --- |
| Global | 0.1 (0.02-2) | 3 (1-6) | 3 (1-8) | 0.1 (0.03-0.1) | 3 (2-5) | 10 (6-17) |
| AFR D^3^ | 0.07 (0-3) | 5 (0-25) | 18 (0-74) | 0 (0-0.01) | 6 (1-16) | 31 (5-105) |
| AFR E | 0.01 (0-1) | 5 (0-28) | 10 (0-42) | 0.03 (0-0.1) | 7 (2-15) | 26 (10-61) |
| AMR A | 0 (0-0.02) | 1 (0-3) | 0.7 (0-3) | 0.08 (0.02-0.2) | 2 (0.5-3) | 4 (2-7) |
| AMR B | 0.07 (0-1) | 0.9 (0-5) | 0.6 (0-3) | 0.2 (0.03-0.7) | 5 (1-12) | 8 (3-6) |
| AMR D | 0.08 (0-2) | 0.9 (0-5) | 0.7 (0-4) | 0.2 (0.05-0.6) | 8 (2-29) | 11 (4-33) |
| EMR B | 0.8 (0-5) | 4 (0-20) | 3 (0-10) | 0.09 (0.01-0.2) | 4 (1-9) | 13 (5-31) |
| EMR D | 0.2 (0-3) | 6 (0-34) | 3 (0-13) | 0.1 (0.02-0.3) | 3 (1-7) | 16 (6-45) |
| EUR A | 0.01 (0-0.04) | 2 (0-4) | 0.5 (0-3) | 0.3 (0.06-0.7) | 2 (0.3-3) | 4 (2-8) |
| EUR B | 0.2 (0-2) | 0.4 (0-3) | 0.1 (0-3) | 0.03 (0-0.1) | 4 (1-9) | 5 (2-12) |
| EUR C | 0.03 (0-0.4) | 0.4 (0-3) | 0.08 (0-2) | 0.05 (0.01-0.2) | 2 (0.5-6) | 3 (1-8) |
| SEAR B | 0.03 (0-6) | 3 (0-17) | 1 (0-15) | 0.02 (0-0.2) | 3 (0.9-7) | 10 (3-31) |
| SEAR D | 0.03 (0-5) | 2 (0-13) | 0.9 (0-15) | 0.01 (0-0.2) | 2 (0.3-4) | 7 (1-26) |
| WPR A | 0.01 (0-4) | 2 (0-4) | 0.8 (0-3) | 0.2 (0.04-0.6) | 2 (0.6-3) | 4 (2-9) |
| WPR B | 0.03 (0-0.5) | 0.8 (0-4) | 0.4 (0-2) | 0 (0-0.01) | 2 (0.8-5) | 4 (2-8) |

^1^ Non-typhoidal *Salmonella enterica*

^2^ Shiga-toxin producing *Escherichia coli*

^3^ Regions are abbreviated as: African Region (AFR), the Region of the Americas (AMR), the Eastern Mediterranean Region (EMR), the European Region (EUR), the South-East Asia Region (SEAR), and the Western Pacific Region (WPR). Subregion labels A-E indicate level of child and adult mortality in ascending order.
