# Supplementary material for: Global disease burden of pathogens in animal source foods, 2010
Source: PLoS One. 2019 Jun 6;14(6):e0216545. doi: 10.1371/journal.pone.0216545 (PMC6553721; doi:10.1371/journal.pone.0216545)
Supplement: S3 Table — (DOCX) [file pone.0216545.s003.docx]

S3 Table. Burden (Disability-Adjusted Life Years per 100,000 population) due to consumption of pork, 2010 (median, 95% uncertainty interval)

|  | *Brucella* spp. | *Campylobacter* spp. | NTS^1^ | STEC^2^ | *Trichinella* spp. | *Taenia solium* | *Toxoplasma gondii* | All hazards |
| --- | --- | --- | --- | --- | --- | --- | --- | --- |
| Global | 0.2 (0.1-7) | 2 (0.8-6) | 6 (2-12) | 0.01 (0.003-0.1) | 0.01 (0.004-0.01) | 41(31-52) | 2(1-3) | 51 (40-67) |
| AFR D^3^ | 0.3 (0-10) | 0.2 (0-12) | 17 (0-82) | 0 (0-0) | 0 (0-0) | 170 (110-283) | 1 (0.1-6) | 200 (129-325) |
| AFR E | 0.04 (0-3) | 0.3 (0-13) | 10 (0-51) | 0 (0-0.03) | 0 (0-0) | 176 (134 -229) | 3 (0.5-8) | 196 (148-261) |
| AMR A | 0 (0-0.03) | 0.3 (0-2) | 1 (0-4) | 0 (0-0.02) | 0.01 (0.01-0.01) | 0.4 (0.3-0.6) | 0.9 (0.2-3) | 3 (1-7) |
| AMR B | 0.09 (0-1) | 0.05 (0-4) | 0.6 (0-3) | 0 (0-0.1) | 0.01 (0.01-0.01) | 25 (19-32) | 4 (1-11) | 32 (24-41) |
| AMR D | 0.1 (0-3) | 0.06 (0-4) | 0.7 (0-4) | 0 (0-0.1) | 0.01 (0.01-0.01) | 69 (51-91) | 6 (1-23) | 78 (60-106) |
| EMR B | 2 (0-11) | 0.1 (0-3) | 2 (0-9) | 0 (0-0.03) | 0.0001 (0-0.0003) | 0 (0-0) | 0.4 (0.04-5) | 6 (2-18) |
| EMR B^4^ | 0.02 (0-0.11) | 0.001(0-0.03) | 0.02(0-0.09) | 0 (0-0.0003) | 0 | 0 | 0.004 (0.0004-0.05) | 0.06 (0.02-0.19) |
| EMR D | 0.7 (0-13) | 0.2 (0-8) | 2 (0-12) | 0 (0-0.02) | 0.0001 (0-0.0003) | 0 (0-0) | 0.4 (0.04-4) | 6 (1-24) |
| EMR D^4^ | 0 | 0 | 0 | 0 | 0 | 0 | 0 | 0 |
| EUR A | 0.01 (0-0.09) | 0.6 (0-3) | 3 (0.08-7) | 0.02 (0-0.2) | 0.04 (0.02-0.07) | 0 (0-0) | 1 (0.3-4) | 5 (2-10) |
| EUR B | 0.3 (0-3) | 0.6 (0-2) | 2 (0.1-6) | 0 (0-0.04) | 0.04 (0.02-0.07) | 0 (0-0) | 2 (0.3-6) | 5 (2-12) |
| EUR C | 0.05 (0-0.6) | 0.6 (0-2) | 2 (0.08-5) | 0.01 (0-0.06) | 0.04 (0.02-0.07) | 1 (0.6-2) | 1 (0.2-5) | 6 (3-11) |
| SEAR B | 0.05 (0-8) | 5 (0-19) | 9 (0-33) | 0.02 (0-0.2) | 0.0007 (0.0002-0.001) | 3 (2-5) | 2 (0.3-6) | 21 (8-52) |
| SEAR D | 0.1 (0-18) | 5 (0-19) | 7 (0-32) | 0.02 (0-0.2) | 0.0004 (0.0002-0.001) | 45 (34-60) | 1 (0.1-5) | 62 (42-99) |
| WPR A | 0.02 (0-4) | 0.3 (0-2) | 1 (0.06-4) | 0 (0-0.1) | 0.004 (0.001-0.007) | 0 (0-0) | 0.8 (0.1-3) | 3 (1-9) |
| WPR B | 0.04 (0-0.8) | 1 (0-4) | 1 (0.01-4) | 0 (0-0.01) | 0.004 (0.001-0.007) | 27 (20-35) | 1 (0.3-4) | 31 (24-40) |

^1^ Non-typhoidal *Salmonella enterica*

^2^ Shiga-toxin producing *Escherichia coli*

^3^ Regions are abbreviated as: African Region (AFR), the Region of the Americas (AMR), the Eastern Mediterranean Region (EMR), the European Region (EUR), the South-East Asia Region (SEAR), and the Western Pacific Region (WPR). Subregion labels A-E indicate level of child and adult mortality in ascending order.

^4^ Scenario analysis, taking very low pork consumption in this region into account. Only in Cyprus and Lebanon is there significant pork consumption in EMR B. These countries represent 1.2% of the population of EMR, hence the DALYs attributable to pork were estimated by adjusting the DALYs / 100,000 in EMR B by a multiplication factor of 0.012. In EMR D, pork consumption is negligible and hence DALYs attributable to pork were set at 0.
