# Supplementary material for: Global disease burden of pathogens in animal source foods, 2010
Source: PLoS One. 2019 Jun 6;14(6):e0216545. doi: 10.1371/journal.pone.0216545 (PMC6553721; doi:10.1371/journal.pone.0216545)
Supplement: S4 Table — (DOCX) [file pone.0216545.s004.docx]

S4 Table. Burden (Disability-Adjusted Life Years per 100,000 population) due to consumption of poultry, 2010 (median, 95% uncertainty interval)

|  | *Campylobacter* spp. | NTS^1^ | *Toxoplasma gondii* | All hazards |
| --- | --- | --- | --- | --- |
| Global | 13 (9-20) | 17 (9-29) | 1 (0.6-2) | 32 (21-46) |
| AFR D^2^ | 37 (13-70) | 104 (7-237) | 1 (0.09-6) | 144 (37-292) |
| AFR E | 34 (11-68) | 59 (2-133) | 1 (0.1-5) | 96 (27-182) |
| AMR A | 5 (1-8) | 2 (0.02-5) | 0.4 (0.05-1) | 7 (3-12) |
| AMR B | 7 (3-14) | 3 (0.1-7) | 1 (0.2-5) | 12 (6-21) |
| AMR D | 8 (3-15) | 4 (0.4-10) | 2 (0.2-11) | 15 (7-27) |
| EMR B | 28 (10-56) | 16 (3-34) | 1 (0.2-6) | 46 (20-82) |
| EMR D | 49 (18-87) | 23 (5-46) | 0.4 (0-5) | 73 (31-124) |
| EUR A | 5 (2-9) | 2 (0.06-7) | 0.5 (0.06-2) | 8 (4-14) |
| EUR B | 3 (1-7) | 2 (0.09-6) | 0.7 (0.09-3) | 7 (2-12) |
| EUR C | 3 (1-6) | 2 (0.05-6) | 0.6 (0.08-2) | 6 (3-11) |
| SEAR B | 10 (0.9-36) | 15 (2-48) | 1 (0.1-4) | 29 (9-69) |
| SEAR D | 9 (0-34) | 12 (0-44) | 1 (0.1-4) | 24 (2-66) |
| WPR A | 5 (2-9) | 1 (0.07-4) | 0.6 (0.05-2) | 7 (3-12) |
| WPR B | 4 (0.9-8) | 2 (0.07-5) | 1 (0.2-3) | 7 (3-13) |

^1^ Non-typhoidal *Salmonella enterica*

^2^ Regions are abbreviated as: African Region (AFR), the Region of the Americas (AMR), the Eastern Mediterranean Region (EMR), the European Region (EUR), the South-East Asia Region (SEAR), and the Western Pacific Region (WPR). Subregion labels A-E indicate level of child and adult mortality in ascending order.
