# Supplementary material for: Global disease burden of pathogens in animal source foods, 2010
Source: PLoS One. 2019 Jun 6;14(6):e0216545. doi: 10.1371/journal.pone.0216545 (PMC6553721; doi:10.1371/journal.pone.0216545)
Supplement: S5 Table — (DOCX) [file pone.0216545.s005.docx]

S5 Table. Burden (Disability-Adjusted Life Years per 100,000 population) due to consumption of small ruminant meats, 2010 (median, 95% uncertainty interval)

|  | *Brucella* spp. | *Campylobacter* spp. | NTS^1^ | STEC^2^ | *Toxoplasma gondii* | All hazards |
| --- | --- | --- | --- | --- | --- | --- |
| Global | 1 (0.5-30) | 3 (2-7) | 5 (1-13) | 0.03 (0.01-0.1) | 3 (2-4) | 13 (8-42) |
| AFR D^3^ | 1 (0.1-37) | 7 (0-22) | 16 (0-105) | 0 (0-0.01) | 6 (1-15) | 36 (8-139) |
| AFR E | 0.2 (0-12) | 7 (0-24) | 9 (0-57) | 0.02 (0-0.06) | 4 (0.9-10) | 24 (7-78) |
| AMR A | 0.06 (0.02-0.6) | 0.4 (0-2) | 0.2 (0-1) | 0.01 (0-0.04) | 0.6 (0.2-2) | 1 (0.6-3) |
| AMR B | 1 (0.2-12) | 1 (0-4) | 0.5 (0-3) | 0.05 (0-0.3) | 2 (0.5-8) | 6 (3-19) |
| AMR D | 1 (0.1-28) | 1 (0-5) | 0.6 (0-3) | 0.06 (0-0.2) | 3 (0.4-13) | 7 (3-40) |
| EMR B | 19 (2-67) | 6 (0-21) | 2 (0-14) | 0.05 (0-0.1) | 8 (3-16) | 39 (17-88) |
| EMR D | 3 (0.4-44) | 11 (0-34) | 3 (0-18) | 0.06 (0-0.2) | 8 (3-15) | 28 (12-77) |
| EUR A | 0.3 (0.06-1) | 0.4 (0-2) | 0.2 (0-1) | 0.05 (0-0.2) | 0.7 (0.09-2) | 2 (0.7-4) |
| EUR B | 3 (0.5-27) | 0.6 (0-3) | 0.2 (0-3) | 0 (0-0.03) | 1 (0.3-3) | 6 (2-30) |
| EUR C | 0.6 (0.05-5) | 0.5 (0-2) | 0.2 (0-3) | 0.01 (0-0.05) | 2 (0.5-5) | 4 (2-10) |
| SEAR B | 0.6 (0-83) | 5 (0-20) | 4 (0-35) | 0.05 (0-0.4) | 2 (0.5-6) | 18 (5-104) |
| SEAR D | 0.5 (0-63) | 3 (0-17) | 3 (0-36) | 0.04 (0-0.4) | 1 (0.2-5) | 14 (2-81) |
| WPR A | 0.5 (0.02-107) | 0.4 (0-2) | 0.3 (0-1) | 0.06 (0-0.3) | 0.8 (0.2-2) | 3 (0.9-109) |
| WPR B | 0.5 (0.07-7) | 0.7 (0-3) | 0.5 (0-3) | 0 (0-0.01) | 2 (0.4-4) | 4 (2-12) |

^1^ Non-typhoidal *Salmonella enterica*

^2^ Shiga-toxin producing *Escherichia coli*

^3^ Regions are abbreviated as: African Region (AFR), the Region of the Americas (AMR), the Eastern Mediterranean Region (EMR), the European Region (EUR), the South-East Asia Region (SEAR), and the Western Pacific Region (WPR). Subregion labels A-E indicate level of child and adult mortality in ascending order.
