# Supplementary material for: Global disease burden of pathogens in animal source foods, 2010
Source: PLoS One. 2019 Jun 6;14(6):e0216545. doi: 10.1371/journal.pone.0216545 (PMC6553721; doi:10.1371/journal.pone.0216545)
Supplement: S6 Table — (DOCX) [file pone.0216545.s006.docx]

S6 Table. Burden (Disability-Adjusted Life Years per 100,000 population) due to consumption of dairy, 2010 (median, 95% uncertainty interval)

|  | *Brucella* spp. | *Campylobacter* spp. | NTS^1^ | *Cryptosporidum* spp. | STEC^2^ | *Mycobacterium* *bovis* | *Toxoplasma gondii* | All hazards |
| --- | --- | --- | --- | --- | --- | --- | --- | --- |
| Global | 1 (0.5-30) | 4 (2-8) | 4 (2-9) | 0.3 (0.1-2) | 0.03 (0.01-1) | 9 (7-12) | 0.04 (0.002-0.5) | 20 (12-48) |
| AFR D^3^ | 1 (0.1-37) | 9 (0.2-26) | 17 (0-73) | 0.4 (0-8) | 0 (0-0.01) | 25 (15-39) | 0.004 (0-4) | 58 (27-143) |
| AFR E | 0.2 (0-12) | 9 (0.4-27) | 10 (0-41) | 0.4 (0-10) | 0.01 (0-0.04) | 34 (21-48) | 0.004 (0-3) | 58 (35-99) |
| AMR A | 0.06 (0.02-0.6) | 0.6 (0-4) | 0.5 (0-2) | 0.01 (0-0.1) | 0.02 (0-0.07) | 0.03 (0.01-0.06) | 0.001 (0-0.5) | 2 (0.3-5) |
| AMR B | 1 (0.2-12) | 2 (0.02-5) | 0.5 (0-2) | 0.05 (0-0.5) | 0.05 (0-0.2) | 0.4 (0.21-0.8) | 0.004 (0-1.9) | 4 (2-16) |
| AMR D | 1 (0.1-28) | 2 (0.05-6) | 0.7 (0-3) | 0.09 (0-0.7) | 0.06 (0-0.2) | 2 (0.8-4) | 0.005 (0-3) | 7 (3-34) |
| EMR B | 19 (2-67) | 7 (0.2-20) | 3 (0.2-10) | 0.1 (0-4) | 0.03 (0-0.09) | 1 (0.5-3) | 0.004 (0-2) | 33 (13-83) |
| EMR D | 3 (0.4-44) | 13 (0.7-33) | 4 (0.2-12) | 0.09 (0-4) | 0.03 (0-0.1) | 13 (6-25) | 0.004 (0-2) | 35 (18-80) |
| EUR A | 0.3 (0.06-1) | 0.7 (0-3) | 0.3 (0-3) | 0.005 (0-0.1) | 0.1 (0-0.3) | 0.08 (0.06-0.1) | 0.002(0-0.7) | 2 (0.6-5) |
| EUR B | 3 (0.5-26) | 1 (0-3) | 0.2 (0-3) | 0.005 (0-0.2) | 0.01 (0-0.05) | 0.6 (0.5-1) | 0 (0-1) | 6 (3-29) |
| EUR C | 0.6 (0.05-5) | 1 (0-3) | 0.2 (0-3) | 0.01 (0-0.2) | 0.02 (0-0.08) | 3 (2-5) | 0.002 (0-1) | 6 (3-11) |
| SEAR B | 0.6 (0-83) | 5 (0-20) | 3 (0-19) | 0.1 (0-4) | 0.03 (0-0.3) | 11 (4-27) | 0.002 (0-1) | 25 (10-111) |
| SEAR D | 0.5 (0-63) | 4 (0-19) | 3 (0-24) | 0.1 (0-5) | 0.02 (0-0.2) | 14 (6-27) | 0.001 (0-0.9) | 27 (11-90) |
| WPR A | 0.5 (0.02-107) | 0.4 (0-3) | 0.4 (0-2) | 0.003 (0-0.1) | 0.05 (0-0.2) | 0.1 (0.08-0.2) | 0.0008 (0-0.6) | 2 (0.5-110) |
| WPR B | 0.5 (0.07-7) | 1 (0-4) | 0.4 (0-2) | 0.01 (0-0.4) | 0 (0-0.01) | 3 (1-5) | 0.001 (0-0.9) | 5 (3-12) |

^1^ Non-typhoidal *Salmonella enterica*

^2^ Shiga-toxin producing *Escherichia coli*

^3^ Regions are abbreviated as: African Region (AFR), the Region of the Americas (AMR), the Eastern Mediterranean Region (EMR), the European Region (EUR), the South-East Asia Region (SEAR), and the Western Pacific Region (WPR). Subregion labels A-E indicate level of child and adult mortality in ascending order.
