# Supplementary material for: Global disease burden of pathogens in animal source foods, 2010
Source: PLoS One. 2019 Jun 6;14(6):e0216545. doi: 10.1371/journal.pone.0216545 (PMC6553721; doi:10.1371/journal.pone.0216545)
Supplement: S7 Table — (DOCX) [file pone.0216545.s007.docx]

S7 Table. Burden (Disability-Adjusted Life Years per 100,000 population) due to consumption of eggs, 2010 (median, 95% uncertainty interval)

|  | NTS^1^ | *Toxoplasma gondii* | All hazards |
| --- | --- | --- | --- |
| Global | 10 (5-19) | 0.01 (0.001-0.2) | 10 (5-19) |
| AFR D^2^ | 64 (0-176) | 0.005 (0-0.5) | 64 (0.01-176) |
| AFR E | 37 (0-99) | 0.004 (0-0.4) | 37 (0.02-99) |
| AMR A | 2 (0.02-6) | 0.001 (0-0.1) | 2 (0.04-6) |
| AMR B | 2 (0-5) | 0.003 (0-0.4) | 2 (0.01-6) |
| AMR D | 3 (0.09-7) | 0.005 (0.0001-0.5) | 3 (0.1-7) |
| EMR B | 10 (1-25) | 0.004 (0-0.4) | 10 (1-25) |
| EMR D | 13 (0.7-33) | 0.004 (0.0001-0.5) | 13 (0.8-33) |
| EUR A | 3 (0-7) | 0.0007 (0-0.1) | 3 (0-7) |
| EUR B | 3 (0.1-8) | 0.002 (0-0.2) | 3 (0.2-8) |
| EUR C | 3 (0.3-7) | 0.002 (0-0.2) | 3 (0.3-7) |
| SEAR B | 5 (0-24) | 0.002 (0-0.3) | 5 (0.01-24) |
| SEAR D | 5 (0-30) | 0.001 (0-0.2) | 5 (0-28) |
| WPR A | 2 (0-5) | 0.0001 (0-0.1) | 2 (0-5) |
| WPR B | 0.9 (0-4) | 0.002 (0-0.2) | 0.9 (0.01-4) |

^1^ Non-typhoidal *Salmonella enterica*

^2^ Regions are abbreviated as: African Region (AFR), the Region of the Americas (AMR), the Eastern Mediterranean Region (EMR), the European Region (EUR), the South-East Asia Region (SEAR), and the Western Pacific Region (WPR). Subregion labels A-E indicate level of child and adult mortality in ascending order.
