# Supplementary material for: Global disease burden of pathogens in animal source foods, 2010
Source: PLoS One. 2019 Jun 6;14(6):e0216545. doi: 10.1371/journal.pone.0216545 (PMC6553721; doi:10.1371/journal.pone.0216545)
Supplement: S8 Table — (DOCX) [file pone.0216545.s008.docx]

S8 Table. Burden (Disability-Adjusted Life Years per 100,000 population) due to consumption of finfish, 2010 (median, 95% uncertainty interval)

|  | NTS^1^ | *Clonorchis* spp. | *Intestinal* *flukes* | *Opisthorchis* spp. | All hazards |
| --- | --- | --- | --- | --- | --- |
| Global | 1 (0.4-3) | 8 (6-9) | 2 (2-3) | 3 (2-3) | 14 (12-17) |
| AFR D^2^ | 5 (0-28) | 0 (0-0) | 0.01 (0-0.04) | 0 (0-0) | 5 (0.01-28) |
| AFR E | 3 (0-14) | 0 (0-0) | 0 (0-0) | 0 (0-0) | 3 (0.02-14) |
| AMR A | 0.04 (0-0.4) | 0 (0-0) | 0.1 (0.04-0.5) | 0 (0-0) | 0.2 (0.06-0.7) |
| AMR B | 0.2 (0-0.8) | 0 (0-0) | 0.06 (0.02-0.2) | 0 (0-0) | 0.3 (0.1-0.9) |
| AMR D | 0.2 (0-1) | 0 (0-0) | 0 (0-0) | 0 (0-0) | 0.2 (0-1) |
| EMR B | 0.8 (0-4) | 0 (0-0) | 0.06 (0.02-0.2) | 0 (0-0) | 0.9 (0.08-4) |
| EMR D | 1 (0-5) | 0 (0-0) | 0.08 (0.03-0.2) | 0 (0-0) | 1 (0.1-5) |
| EUR A | 0 (0-2) | 0 (0-0) | 0.03 (0.01-0.09) | 0.07 (0.02-0.3) | 0.2 (0.03-2) |
| EUR B | 0.05 (0-0.7) | 0 (0-0) | 0.05 (0.02-0.2) | 0.05 (0.01-0.3) | 0.2(0.04-0.9) |
| EUR C | 0.04 (0-0.7) | 0.04 (0.03-0.04) | 0.09 (0.03-0.2) | 0.9 (0.6-1) | 1 (0.8-2) |
| SEAR B | 0.8 (0-6) | 0.01 (0-0.04) | 0.2 (0.1-0.5) | 40 (32-50) | 41 (33-52) |
| SEAR D | 0.7 (0-6) | 0.04 (0.01-0.2) | 0.1 (0.03-0.4) | 0.4 (0.1-2) | 1 (0.3-7) |
| WPR A | 0.04 (0-0.6) | 0.05 (0.01-0.2) | 1 (0.9-2) | 0 (0-0) | 2 (1-2) |
| WPR B | 0.1 (0-0.7) | 31 (26-38) | 9 (7-11) | 3 (2-4) | 44 (37-51) |

^1^ Non-typhoidal *Salmonella enterica*

^2^ Regions are abbreviated as: African Region (AFR), the Region of the Americas (AMR), the Eastern Mediterranean Region (EMR), the European Region (EUR), the South-East Asia Region (SEAR), and the Western Pacific Region (WPR). Subregion labels A-E indicate level of child and adult mortality in ascending order.
