# Supplementary material for: Global disease burden of pathogens in animal source foods, 2010
Source: PLoS One. 2019 Jun 6;14(6):e0216545. doi: 10.1371/journal.pone.0216545 (PMC6553721; doi:10.1371/journal.pone.0216545)
Supplement: S9 Table — (DOCX) [file pone.0216545.s009.docx]

S9 Table. Burden (Disability-Adjusted Life Years per 100,000 population) due to consumption of shellfish, 2010 (median, 95% uncertainty interval)

|  | NTS^1^ | *Paragonimus* spp. | All hazards |
| --- | --- | --- | --- |
| Global | 1 (0.3-4) | 15 (11-21) | 16 (12-23) |
| AFR D^2^ | 4 (0-29) | 0.03 (0.01-0.08) | 3 (0.03-29) |
| AFR E | 2 (0-13) | 0.01 (0-0.02) | 2 (0.01-13) |
| AMR A | 0.08 (0-0.6) | 0.04 (0-0.6) | 0.2 (0.02-1) |
| AMR B | 0.1 (0-0.9) | 0.04 (0.01-0.1) | 0.2 (0.03-1) |
| AMR D | 0.2 (0-1) | 53 (38-73) | 54 (38-74) |
| EMR B | 0.5 (0-3) | 0 (0-0) | 0.5 (0-3) |
| EMR D | 0.7 (0-5) | 0.02 (0.01-0.07) | 0.8 (0.03-5) |
| EUR A | 0.09 (0-0.8) | 0 (0-0) | 0.09 (0-0.8) |
| EUR B | 0.1 (0-1) | 0 (0-0) | 0.1 (0-1) |
| EUR C | 0.1 (0-0.9) | 0.03 (0.01-0.1) | 0.2 (0.03-1) |
| SEAR B | 0.8 (0-5) | 0.05 (0.01-0.5) | 0.9 (0.04-5) |
| SEAR D | 0.8 (0-9) | 0.06 (0.02-0.2) | 0.9 (0.04-9) |
| WPR A | 0.06 (0-0.10) | 0.05 (0.02-0.2) | 0.2 (0.03-1) |
| WPR B | 0.1 (0-0.7) | 60 (43-83) | 61 (42-83) |

^1^ Non-typhoidal *Salmonella enterica*

^2^ Regions are abbreviated as: African Region (AFR), the Region of the Americas (AMR), the Eastern Mediterranean Region (EMR), the European Region (EUR), the South-East Asia Region (SEAR), and the Western Pacific Region (WPR). Subregion labels A-E indicate level of child and adult mortality in ascending order.
